# Exceptional antineoplastic activity of a dendritic-cell-targeted vaccine loaded with a *Listeria* peptide proposed against metastatic melanoma

**DOI:** 10.18632/oncotarget.7806

**Published:** 2016-03-01

**Authors:** Ricardo Calderon-Gonzalez, Lucia Bronchalo-Vicente, Javier Freire, Elisabet Frande-Cabanes, Lidia Alaez-Alvarez, Javier Gomez-Roman, Sonsóles Yañez-Diaz, Carmen Alvarez-Dominguez

**Affiliations:** ^1^ Group of Genomics, Proteomics and Vaccines, Marqués de Valdecilla Research Institute (IDIVAL), Santander, Spain; ^2^ Dermatology Department, Marqués de Valdecilla University Hospital (HUMV), Santander, Spain; ^3^ Pathological Anatomy Department, Marqués de Valdecilla University Hospital (HUMV), Santander, Spain

**Keywords:** dendritic cells, Listeria peptides, vaccines, melanoma, immunotherapy

## Abstract

Vaccination with dendritic cells (DCs) is proposed to induce lasting responses against melanoma but its survival benefit in patients needs to be demonstrated. We propose a DC-targeted vaccine loaded with a *Listeria* peptide with exceptional anti-tumour activity to prevent metastasis of melanoma. Mice vaccinated with vaccines based on DCs loaded with listeriolysin O peptide (91–99) (LLO_91–99_) showed clear reduction of metastatic B16OVA melanoma size and adhesion, prevention of lung metastasis, enhanced survival, and reversion of immune tolerance. Robust innate and specific immune responses explained the efficiency of DC-LLO_91–99_ vaccines against B16OVA melanoma. The noTable features of this vaccine related to melanoma reduction were: expansion of immune-dominant LLO_91–99_-specific CD8 T cells that helped to expand melanoma-specific CD8^+^ T cells; high numbers of tumour-infiltrating lymphocytes with a cytotoxic phenotype; and a decrease in CD4^+^CD25^high^ regulatory T cells. This vaccine might be a useful alternative treatment for advanced melanoma, alone or in combination with other therapies.

## INTRODUCTION

Human melanoma is a malignant tumour of melanocytes and an aggressive skin cancer that has registered a 3% increase in annual incidence in Northern Spain [1]. Melanoma is one of the most rapidly growing cancers worldwide but yet there is no satisfactory treatment except for surgery, either in the early stages or when it has advanced to metastatic disease. Pharmacological treatment with small molecule inhibitors such as vemurafenib leads to resistance and has major cutaneous effects, but fails to induce lasting responses, which has turned the focus to immunotherapy. Classical immunotherapy agents such as interleukin (IL)-2 [2] or interferon (IFN)-α induce durable responses but the survival benefit is low. New immunomodulatory antibodies such as ipilimumab and nivolumab that block T-cell-negative regulators cytotoxic T-lymphocyte-associated antigen-4 and programmed death-1, respectively, cause some side effects, such as high toxicity and activation of autoreactive T cells [3]. In this context, dendritic cells (DCs) are pivotal cells of the immune system with high capacity to induce T-cell immunity, and are used as vaccines to increase host resistance to melanoma [4, 5]. However, the lack of immunodominant melanoma antigens triggering potent cytotoxic T-cell responses, induction of immune-suppressor T cells known as regulatory T (Treg) cells in melanoma patients, and a limited beneficial effect on survival, has dampened widespread use of DC-based vaccines as melanoma immunotherapy [6, 7].

Activation of DCs as cancer vaccines can be performed *in vitro* [6, 7] or *in vivo* as in the case of different *Listeria monocytogenes* strains [8–11]. The exceptional adjuvant properties of the main *Listeria* antigen, listeriolysin O (LLO), such as activation of DCs, stimulation of potent cytotoxic T cells, disabling of the immune unresponsiveness against tumours, and enhancement of T helper (Th)1-dominant immune responses, explains the success of attenuated *Listeria* as an anti-tumour vaccine [12, 13]. Another adjuvant property of LLO that is useful for cancer therapy is its ability to target to murine and human melanoma cells and transform them into DCs, causing melanoma regression [10]. Finally, the immune-dominant response of LLO peptide 91–99 (LLO_91–99_) when presented to cytotoxic T cells, affecting the immune response to other antigens, is relevant for cancer and prophylactic vaccines [14–16]. LLO_91–99_ and glyceraldehyde-3-phosphate dehydrogenase (GAPDH), GAPDH peptide 1–22 (GAPDH_1–22_) and GAPDH_1–15_ were used with success in DC vaccines for listeriosis as they induce strong cytotoxic-T-cell responses and DC activation [17–19], which are both useful properties for cancer vaccines.

Adjuvant properties of bacterial antigens to improve cancer therapy is not a new phenomenon, and Coley observed that a patient with neck cancer recovered after infection with erysipelas, which initiated the use of bacteria and their toxins to treat end-stage cancer [20]. However, pathogenicity and toxicity are important concerns limiting the broad clinical application of bacteria and their toxins as anti-cancer agents for immune-compromised patients.

In our search for safe immunotherapy for advanced melanoma, we used experimental DC vaccines loaded with different peptides of LLO or GAPDH virulence factors of *Listeria*, to examine their anti-tumour potential against metastatic B16OVA melanoma. The use of peptides of LLO or GAPDH, instead of attenuated pathogens or recombinant LLO, a pore-forming cytolysin, such as other groups have proposed [12–14], supposes that our therapy is safe and non-toxic.

## RESULTS AND DISCUSSION

The objective of this study was to identify a highly stimulatory DC vaccine with a survival benefit that prevented advanced melanoma. Since 2001, patients with advanced melanoma enrolled in phase II clinical studies have been administered autologous DC loaded with melanoma lysates or a cocktail of melanoma peptides, and showed some clinical benefit [4, 5, 7]. However, efforts should be made to implement these promising therapies since a robust CD8^+^ T-cell response that correlates with tumour regression is not always obtained and, more significantly, the number of patients with increased survival is small. According to the American Joint Committee on Cancer in 2009, advanced melanoma corresponds to any size of tumour, spreading to lymph nodes and other organs and distant metastasis [21]. Advanced melanoma is characterized also by severe immune tolerance, explained in part by low percentages of tumour peptides or poor immunogenicity of melanoma antigens.

### B16OVA model of metastatic melanoma and DC vaccination

**Figure 1:**
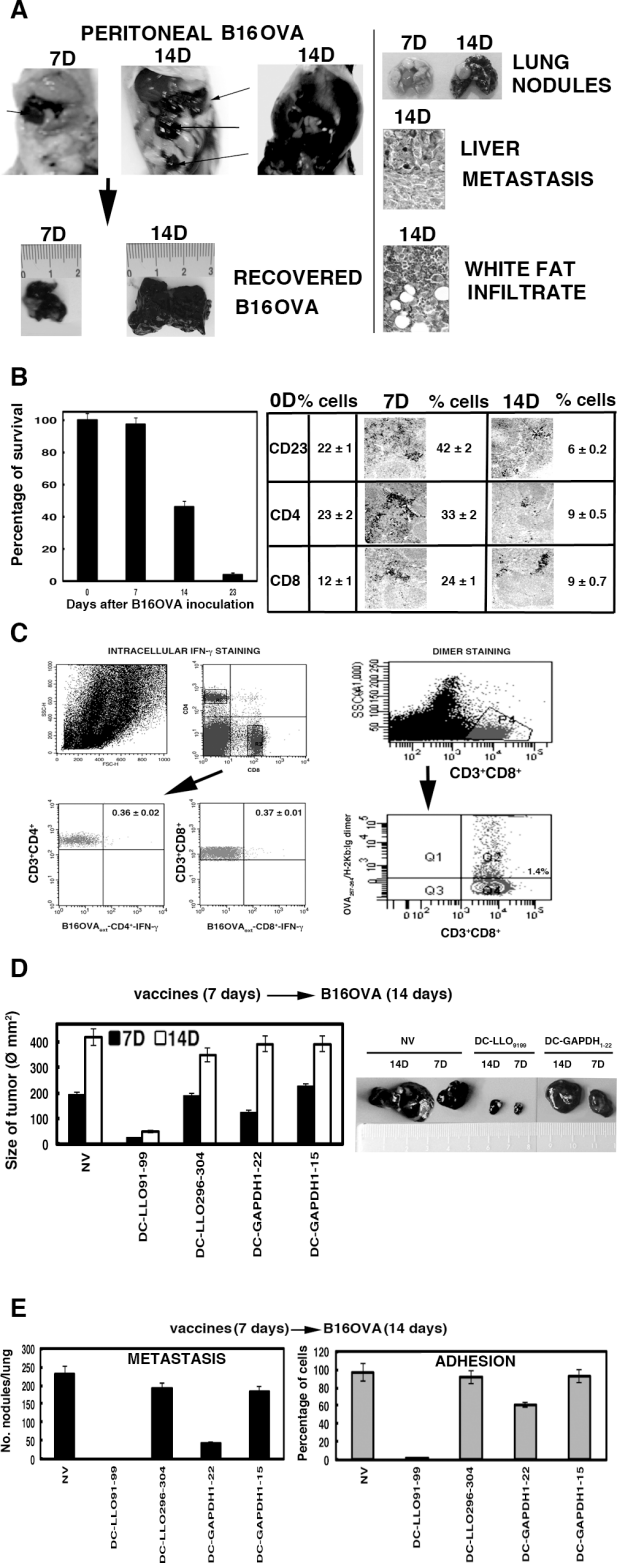
B16OVA model of metastatic melanoma and DC vaccination (**A**) B16OVA was transplanted intraperitoneally into mice for 7 (7D) or 14 (14D) days. Melanoma was recovered post-transplantation and measured immediately with a calliper (lower images) (*n =* 10). We also quantified post-transplantation the number of metastases as visual nodules in the lungs or after histological analysis of the peritoneum and liver (right images). (**B**) Mice were transplanted with B16OVA for 0, 7, 14 or 23 days (*n =* 10/time) (left plot) and the number of surviving mice counted. Results are expressed as the percentages of surviving mice (*P* < 0.05). At the end of transplantation, mice were killed and spleens immediately removed and processed for histological analysis (right images) or cell populations by FACS. Results are expressed as the mean of percentages of positive cells ± SD (right values plot) (*P* < 0.05). (**C**) Mice were transplanted with B16OVA for 14 days, killed, and spleens were removed (*n =* 5). Intracellular cytokine staining was performed immediately in isolated spleen homogenates stimulated with B16OVA extract (B16OVA_ext_) in the presence of brefeldin A (left histograms showing the percentages of B16OVA_ext_-CD4^+^ and B16OVA_ext_-CD8^+^ and IFN-γ producers). Frequencies of CD8^+^-OVA_257–264_ were examined using dimers of recombinant dimeric H-2K^b^: Ig fusion protein loaded with OVA_257–264_ peptide (right plots) (*P* < 0.05). (**D**) Mice were vaccinated with DC-LLO_91–99_, DC-LLO_296–304_, DC-GAPDH_1–15_ or DC-GAPDH_1–22_ vaccines or left unvaccinated (NV) for 7 days. Mice were transplanted with B16OVA for 7 (7D, black bars) or 14 (14D, white bars) days and killed. Size of recovered melanoma was measured with a calliper (Ø mm) (*n =* 5). (**E**) Mice were vaccinated with different DC vaccines for 7 days and transplanted with B16OVA for 14 days. Post-transplantation, we quantified the number of melanoma metastases in the lungs (left plot) and adherence activity of recovered melanoma. Results are expressed as the mean of lung metastases ± SD (left plot) or the percentage of cells adhered to plates (right plot) (*P* < 0.05).

Here, we report the exceptional antineoplastic activity of an immune-dominant peptide of the human pathogen *L. monocytogenes*, LLO_91–99_, incorporated in a DC vaccine against experimental B16OVA melanoma. We provide evidence that this immunotherapy prevents adhesion, dissemination and metastasis of B16OVA murine melanoma, and induces robust innate and specific immune responses to *Listeria* and melanoma. To avoid the low immunogenicity of melanoma antigens, we used murine melanoma B16OVA, a cell line of B16-F10 melanoma cells expressing chicken ovalbumin, an antigen that induces robust CD4^+^ and CD8^+^ T-cell responses [22, 23]. We inoculated B16OVA melanoma into the peritoneum of mice, because they induce carcinomatous peritonitis that allows analysis of tumour growth as well as dissemination and metastasis to the liver and lungs. Subcutaneous and intravenous models of B16OVA do not allow analysis of melanoma dissemination [10, 24, 25]. B16OVA growth in the peritoneum initiates as a single tumour of 12–15 mm at 7 days (Figure 1A), disseminating to several tumours at 14 days of 30–35 mm size in half of the mice (Figure 1A) or colonizing the whole peritoneum in the other half (Figure 1A). These results show that at 14 days, melanoma has already disseminated in the peritoneum. B16OVA melanoma also metastasizes to the liver and lungs at 14 days and strongly infiltrates the peritoneal white adipose tissue (Figure 1A).

Similar to advanced melanoma [3, 21], we found that only 25% of mice survived at 14 days after inoculation with B16OVA cells and only 3% survived at 23 days (Figure 1B). We noticed the deterioration of innate immune responses in the spleen by a decreased percentage of CD23^+^ DCs, from 42% to 6% (Figure 1B) and reductions of CD56^+^ natural killer (NK) cells and CD68^+^ macrophages, from 22%–26% to 8.5%–10% (data not shown). The significant decrease in the percentage of CD4^+^ (from 33% to 9%) and CD8^+^ T cells (from 24% to 9%) (Figure 1B) also suggested a decline in specific immune responses. We confirmed the specific immune tolerance by the strong reduction in Th1 cytokines, monocyte chemoattractant protein-1, tumour necrosis factor (TNF)-α and IFN-γ (Table 1), low 0.37% percentages of B16OVA extract-specific CD8^+^ T cells (Figure 1C) and low (1.4%) frequencies of melanoma OVA_257–264_-specific CD8^+^ T cells using dimers of MHC-I molecules and peptides (Figure 1C) [23]. Therefore, we proposed B16OVA peritoneal growth at 14 days as a model of advanced melanoma.

**Table 1:** Proinflammatory cytokine production of B16OVA transplantation model

^a^Cytokine	Control	B16OVA-7D	B16OVA-14D
**MCP-1**	6.44 ± 0.1	328 ± 1.5	13.4 ± 1.5
**TNF-**α	5.4 ± 0.1	11.39 ± 0.2	1.61 ± 0.1
**IFN-γ**	0.98 ± 0.1	91.9 ± 1.2	0.57 ± 0.1
**IL-6**	0.89 ± 0.1	12.8 ± 1.2	9.69 ± 0.5
**IL-10**	0 ± 0	13.8 ± 0	51.52 ± 0.5
**IL-12**	1.2 ± 0.1	0 ± 0	0 ± 0

a Levels of proinflammatory cytokines analysed in sera of mice transplanted intraperitoneally or not (controls) with B16OVA melanoma for 7 (7D) or 14 (14D) days. Results are expressed as cytokine concentration (pg/ml of mean ± SD, *P* < 0.05).

### DC-LLO_91–99_ vaccines showed exceptional antineoplastic activity

LLO is an immune-dominant antigen from *Listeria* widely used in cancer therapy, because it induces effector CD8^+^ T cells that are localized within the tumour and shows efficient adjuvant properties [26, 27]. Previous observations revealed that LLO is responsible for effective therapy against melanoma, inducing immune-dominant CD8^+^ T-cell responses to melanoma antigens [10]. DC-LLO_91–99_ anti-*Listeria* vaccines induce strong cytotoxic T-cell responses [15, 17–19], therefore, we tested DC-LLO_91–99_ vaccines as melanoma immunotherapy. We also included in our study other immunodominant *Listeria* peptides that induced strong CD8^+^ T-cell responses such as GAPDH_1–15_, GAPDH_1–22_ and LLO_296–304_ [15, 16, 18, 19]. We vaccinated mice in the peritoneum with a single dose of DC-LLO_91–99_, DC-LLO_296–304_, DC-GAPDH_1–15_ or DC-GAPDH_1–22_ formulations and 7 days later, inoculated B16OVA melanoma into the peritoneum. Mice vaccinated with DC-LLO_91–99_ presented 10-fold and 30-fold lower tumour sizes at 7 and 14 days, respectively, than non-vaccinated (NV) mice (Figure 1D). We observed that vaccination with a single dose of DC-LLO_91–99_ decreased the tumour size with time, 10–30-fold, suggesting impairment in melanoma dissemination. Dissemination depends on the ability of the tumours to adhere to the peritoneal mesothelium [24, 25]. Adhesion also affects the ability of tumours to migrate to distant organs and generate metastases. DC-LLO_91–99_ vaccines prevented lung metastasis and adhesion (Figure 1E). The strong antiadhesive activity of DC-LLO_91–99_ vaccines correlated with 2–3-fold reductions in the surface expression of two b2-integrins, CD11b and CD11c, involved in adherence and cell migration (Table 2, MEL-markers). In contrast, DC-GAPDH_1–22_ vaccines showed three- and twofold lower melanoma sizes at 7 and 14 days, respectively (Figure 1D) and fivefold reduction in the number of metastases (Figure 1E). DC-GAPDH_1–22_ vaccines did not show clear antiadhesive activity since adhesion was only 35% decreased (Figure 1E) and expression of CD11b and CD11c was similar to that in NV mice (Table 2). We conclude that DC-GAPDH_1–22_ vaccines showed some anti-melanoma properties. DC-LLO_296–304_ and DC-GAPDH_1–15_ vaccines did not affect melanoma size, dissemination, metastasis or adhesion (Figure 1D, 1E); therefore, we discarded them as melanoma therapies.

**Table 2:** Prognostic factors in recovered B16OVA melanoma of DC-LLO_91–99_-vaccinated mice

Vaccines^a^			
Melanoma markers^c^	NV	DC-LLO_91–99_	DC-GAPDH_1–22_
CD11c^+^	25 ± 0.1	12 ± 0.1	20 ± 0.2
CD11b^+^	26 ± 0.1	9 ± 0.1	27 ± 0.1
F4/80^+^	3.9 ± 0.1	4.4 ± 0.2	4.1 ± 0.3
CD14^+^	2 ± 0.1	9.4 ± 0.2	2.5 ± 0.3
MHC-II^+^	53 ± 0.1	9 ± 0.2	60 ± 0.3
H-2Kb^+^	73 ± 0.1	98 ± 0.2	71 ± 0.3
CD83^+^CD86^+^	3 ± 0.1	21 ± 0.2	3.1 ± 0.3
**TIL markers**^b^	**TIL markers**	**TIL markers**	**TIL markers**
CD4^+^CD25^−^	1.1 ± 0.01	5.4 ± 0.1	1.2 ± 0.1
CD4^+^CD25^high^	0.54 ± 0.01	0.1 ± 0.01	0.50 ± 0.01
CD8^+^	1.3 ± 0.01	9.8 ± 0.01	1.2 ± 0.2
CD14^+^	2 ± 0.01	9.1 ± 0.01	2.3 ± 0.2
CD11b^+^	13 ± 0.01	26 ± 0.01	12 ± 0.2
CD49b^+^	0.3 ± 0.01	6.1 ± 0.01	0.2 ± 0.2
**Mitotix index**^d^	2 ± 0.01	0.1 ± 0.02	1.9 ± 0.02

a Melanoma recovered from peritoneum of mice vaccinated with a single dose of DC-LLO_91–99_ or DC-GAPDH_1–22_ vaccine for 7 days or NV mice, transplanted for 14 days with B16OVA (*n =* 5).

b Cell surface markers analysed by FACS in recovered melanoma from a. Results are expressed as percentages of positive cells compared to total melanoma cells (*P* < 0.05).

c Cell surface markers in TILs of recovered melanoma from a. Results are expressed as percentages of positive cells compared to total TILs (mean ± SD of triplicate samples, *P* < 0.005).

d Mitotic index calculated as the ratio of the number of B16OVA in culture at time 0 h versus the number of cells at 16 h (mean ± SD) (*P* < 0.05).

**Figure 2:**
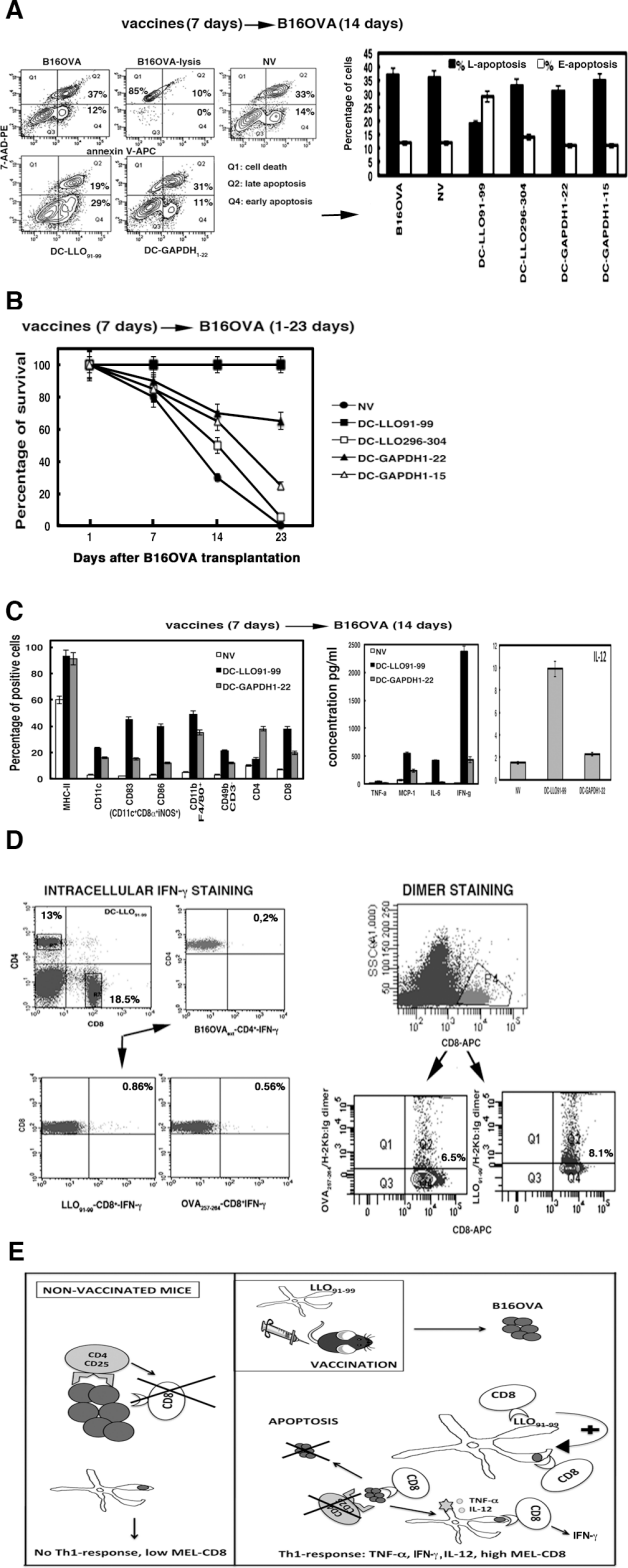
(A) Early and late apoptosis examined in NV or DC-vaccinated mice and transplanted with B16OVA for 14 days (14D) Apoptosis was analysed by FACS after double staining with 7-AAD (7-AAD-PE) and annexin V (annexin V-APC) and expressed as percentages. Q2 area shows late apoptotic cells, and Q4 area shows early apoptotic cells. (**B**) Mice were vaccinated with different DC vaccines or left unvaccinated (NV) for 7 days (*n* = 10). All mice were transplanted with B16OVA and the number of surviving animals was counted at 1, 7, 14 or 23 days. All groups of mice were compared to the NV group. Results correspond to the mean ± SD number of surviving mice (*P* < 0.05). (**C**) Left plot shows spleen cell populations analysed by FACS that corresponded to NV mice or mice vaccinated with DC-LLO_91–99_ or DC-GAPDH_1–22_ for 7 days and transplanted with B16OVA for 14 days (14D). Results expressed as percentages of positive cells ± SD. Right plots show cytokines levels from mice sera, expressed as cytokine concentrations (pg/ml of mean ± SD, *P* < 0.05). (**D**) Intracellular IFN-γ staining of spleens from DC-LLO_91–99_-vaccinated mice and stimulated with OVA_257–264_ or LLO_91–99_ peptides in the presence of brefeldin A (left lower plots). Right plots show the frequencies of CD8^+^–OVA_257–264_ or CD8^+^-LLO_91–99_ using dimers of recombinant H-2K^b^: Ig fusion protein loaded with peptides. Results are the mean ± SD (*P* < 0.05). (**E**) Model of action of DC-LLO_91–99_ vaccines against advanced melanoma.

A major prognostic factor of melanoma survival is the mitotic index of melanoma cells because therapy causing melanoma apoptosis also causes tumour regression [28, 29]. DC-LLO_91–99_ vaccinations showed a 20-fold reduction in the mitotic indexes of recovered melanoma (Table 2), suggesting major alterations to their cell cycle because of apoptosis or necrotic death. An increase in late apoptosis corresponds to necrotic death, while increases in early apoptotic ratios indicates programmed cell death by different stimuli such as cytokines or the presence of tumour-infiltrating lymphocytes (TILs). DC-LLO_91–99_ vaccines eradicated melanoma by programmed cell death and not by necrotic death, as they induced 2.6-fold increases in early apoptosis (Figure 2A). We observed no variation in mitotic indexes or apoptosis of melanoma recovered from mice vaccinated with DC-GAPDH_1–22_ vaccines (Table 2, Figure 2A).

Next, we examined the effect of DC vaccines on mouse survival. We vaccinated mice with DC vaccines for 7 days and transplanted them with B16OVA melanoma for 1, 7, 14 or 23 days. We established groups of mice (*n* = 10) and compared each group with NV mice. DC-LLO_91–99_ vaccines induced 100% survival (Figure 2B), DC-GAPDH_1–22_ vaccines 65% survival (Figure 2B), DC-GAPDH_1–15_ vaccines 25% survival (Figure 2B) and DC-LLO_296–304_ vaccines only 5% survival (Figure 2B).

### DC-LLO_91–99_ vaccines induced melanoma-specific immune responses and reverted melanoma immune tolerance

DC-LLO_91–99_ vaccines showed exceptional antineoplastic activity, thus, we also investigated the immune responses that they induced. A single dose of DC-LLO_91–99_ vaccine for 7 days followed by B16OVA transplantation for 14 days induced robust innate immune responses in the spleen, with high percentages of NK cells of the tumourigenic phenotype CD3^−^CD49b^+^, DC phenotypes involved in tumour elimination CD8α^+^CD11c^+^CD83^+^CD86^+^iNOS^+^MHC-II^+^, and CD11b^+^ macrophages (Figure 2C). Consequently, DC-LLO_91–99_ immunotherapy also produced high levels of IFN-γ and IL-12 Th1 cytokines (Figure 2C) and increased the percentages of CD8^+^ T cells, while CD4^+^ T cells were not affected (Figure 2C). In contrast, DC-GAPDH_1–22_ vaccination followed by B16OVA transplantation failed to stimulate the immune responses (Figure 2C). DC-LLO_91–99_ vaccination followed by B16OVA transplantation resulted in expansion of LLO_91–99_-specific CD8^+^ T cells (Figure 2D), verified by high 8.1% frequencies of LLO_91–99_-specific CD8^+^ T cells producing IFN-γ (Figure 2D). However, DC-LLO_91–99_ immune-therapy for B16OVA melanoma also induced expansion of melanoma OVA_257–264_-specific CD8^+^ T cells (Figure 2D), confirmed by sixfold increased frequency of melanoma-specific CD8^+^ T cells producing IFN-γ (Figure 2D). Therefore, LLO_91–99_ immune-dominant CD8^+^ T-cell responses enhanced melanoma-specific CD8^+^ T cells, measured as OVA_257–264_ specific CD8+ T cells since B16OVA are transfected with OVA. This immune-dominance of CD8^+^ T-cell responses elicited by DC-LLO_91–99_ vaccination bypassed the unresponsiveness of metastatic melanoma.

How can DC-LLO_91–99_ vaccinations break melanoma immune tolerance? The populations of TILs obtained from the recovered melanoma after DC-LLO_91–99_ vaccinations provided insights into this question because we detected eightfold higher percentage of CD8^+^ T cells in melanoma TILs from DC-LLO_91–99_-vaccinated mice than from NV mice (Table 2). We detected that all CD4^+^CD25^+^ T cells in TILs were also positive for the classical T_reg_ marker FoxP3 (data not shown) and considered this phenotype as Treg. We observed fourfold higher percentage of CD4^+^CD25^−^ T cells and 2.4-fold reductions in the percentages of CD4^+^CD25^high^ Treg cells in melanoma TILs from DC-LLO_91–99_-vaccinated mice (Table 2). Other cells involved in tumour immunity also showed enhanced numbers such as CD11b^+^CD14^+^ monocytes/macrophages or CD49b^+^ NK cells (Table 2). We conclude that the ability of LLO_91–99_ peptides to suppress T_reg_ cell function, [30] is maintained in DC-LLO_91–99_ vaccines. Therefore, these immune-therapies induced the migration of lymphocytes to TILs with tumouricidal activity, such as CD8^+^ T cells, CD49b^+^ NK cells or CD11b^+^CD14^+^ monocytes, while reducing the number of immune inhibitory cells such as CD4^+^CD25^high^ cells, thus helping to revert melanoma immune unresponsiveness [31]. Vaccinations with DC-GAPDH_1–22_ showed similar immune cell populations in TILs compared with NV mice (Table 2). Therefore, we discarded DC-GAPDH_1–22_ vaccinations as melanoma immune-therapies.

Does DC-LLO_91–99_ vaccinations provoke changes in melanoma that contribute to immune enhancement? We previously reported that *Listeria* therapy of melanoma transformed tumour cells into DCs [10]. When we analysed cell surface markers of melanoma recovered from DC-LLO_91–99_-vaccinated mice, we detected increased percentages of several DC markers such as CD14^+^, Toll-like receptor (TLR)2 accessory molecule that binds to LLO [32], and activation markers such as double-positive CD83^+^CD86^+^ cells. We also observed that the percentages of H-2K^b^ MHC class I molecules increased, while the percentages of IA^b^ MHC class II molecules were reduced by fivefold (Table 2). Therefore, DC-LLO_91–99_ vaccinations mimicked most of the effects observed with *Listeria* on the transformation of melanoma cells into DCs. This transformation into DCs might also increase CD8^+^ and decrease CD4^+^ T-cell responses. However, it is also possible that DC-LLO_91–99_ vaccination before B16OVA transplantation induces re-programming of DC antigen presentation, increasing melanoma-specific as well as LLO-specific CD8^+^ T-cell interactions and decreasing DC interactions with Treg cells (CD4^+^CD25^+^FoxP3^+^), which is responsible for immune tolerance to melanoma antigens. DC presentation of melanoma antigens in NV mice and B16OVA transplantation triggered a high proportion of Treg cells (CD4^+^CD25^+^FoxP3^+^) that explains the low induction of melanoma-specific CD8^+^ T cells. In this way, DC-LLO_91–99_ vaccinations increased the positive signals between DC and T cells, promoting stronger anti-melanoma immune responses, and controlling tumour growth and dissemination.

In summary, we propose the following molecular model of action of DC-LLO_91–99_ vaccines against advanced melanoma (Figure 2E). In NV and B16 transplanted mice, there is no increase in the percentages of splenic CD83^+^CD86^+^ activated DCs or any change in cell surface markers of melanoma. This scenario seems to expand TILs in melanoma with the CD4^+^CD25^high^ immunosuppressor phenotype that blocks the immune response of melanoma-specific CD8^+^ T cells and abrogates any Th1 cytokine response, allowing the tumour to grow exponentially (Figure 2E). These observations indicate that DC immune responses in NV and melanoma-transplanted mice deliver negative signals to T cells that explain the low percentages of melanoma-specific CD4^+^ and CD8^+^ T cells. DC-LLO_91–99_ vaccination increases expression of co-signalling molecules such as CD83 or CD86 and other co-stimulatory molecules involved in TLR signalling such as CD14 in spleen-activated DCs and melanoma, which re-programmes DCs and melanoma antigen presentation. In this regard, melanoma and splenic DCs present higher expression of MHC class I molecules and significantly reduced expression of MHC class II molecules, driving a Th1 response with high levels of IFN-γ and IL-12. This re-programming causes an increase in the percentages of LLO- and melanoma-specific CD8^+^ T cells, and a decrease in the percentage of melanoma-specific CD4^+^ T cells (Figure 2D, Table 2). Regression of melanoma is also helped by the decreased percentage of negative Treg (CD4^+^CD25^+^FoxP3^+^) cells (Table 2) [33]. These two important changes in the immune response caused by this immune-therapy appeared to induce melanoma apoptosis and promote survival of the mice.

We foresee several scenarios for the future use of DC-LLO_91–99_ vaccinations for advanced melanoma. First, it might function to pre-condition the vaccine site with a potent recall antigen such as LLO_91–99_, similar to tetanus toxoid DC vaccines against glioblastoma [34]. Second, DC-LLO_91–99_ vaccination can be used to increase the positive signals between DC and T cells, promoting stronger anti-melanoma immune responses alone or in combination with other immunotherapies [35, 36]. We suggest that vaccines against pathogens such as *Listeria*, based DC-LLO_91–99_ vaccines or the recently reported tetanus vaccine [34], might help to redesign anti-tumour therapies and prepare the immune system.

## MATERIALS AND METHODS

### Cells, recombinant proteins, peptides and adjuvants

B16OVA melanoma was a gift from I. Melero (CIMA, Navarra University, Pamplona, Spain) and was B16-F10 murine melanoma transfected to express chicken ovalbumin (OVA), as described previously [22, 37]. GAPDH_1–22_, GAPDH_1–15_, LLO_91–99_, LLO_296–304_, LLO_190–201_ and OVA_257–264_ peptides were synthesized in Centro Nacional de Biotecnología (CSIC, Madrid), followed by HPLC and mass spectrometry using a MALDI-TOF Reflex^™^ IV mass spectrometer. Peptide purity was > 95% after HPLC. B16OVA cells (2 × 10^9^) were homogenized as described previously [10], precipitated with TCA, and washed with PBS to obtain a B16OVA extract (B16OVA_ext_) (1 mg/ml). Advax^™^ adjuvant was supplied by N. Petrovsky (Vaxine, Adelaide, Australia) and was mixed with peptides immediately before *ex vivo* DC loading (see below) [19].

### Isolation of differentiated DCs

Differentiated DCs were prepared as described previously [17–19]. Bone-marrow-derived DCs were obtained from femurs of 8–12-week-old female C57BL/6 mice and differentiated in culture with granulocyte–macrophage colony-stimulating factor (30 ng/ml) for 5 days, detached, and positively selected with anti-mouse CD11c-coated magnetic beads and magnetic MACS^™^ separation columns (Miltenyi Biotech Inc., Auburn, CA, USA). CD11c^+^-DCs (immature DCs) were analysed by fluorescence-activated cell sorting (FACS) to assure quality and MHC-II^+^CD11c^+^CD40^+^CD11b^low^CD86^−^F4/80^−^Gr-1^−^ phenotypes.

### Preparation of DCm-peptide vaccines

GAPDH_1–22_, GAPDH_1–15_, LLO_91–99_ or LLO_296–304_ (50 μg/ml) was mixed with 50 μg/ml Advax. Positively selected immature DCs were *ex vivo* loaded with each peptide–adjuvant mixture for 16 h in culture to achieve at least 35%–40% of mature CD11c^+^-DCs (DCm-peptide) with MHC-II^high^CD11c^+^CD40^+^CD86^+^ phenotypes, as previously reported [18, 19]. We also collected supernatants for cytokine analysis by FACS and DCm-peptide was washed extensively before *in vivo* immunization. DCm-peptide vaccines produced significant levels of TNF-α (713 ± 32 pg/ml) and low levels of IL-12 (5 ± 0.6 pg/ml). However, after inoculation into mice they induced high levels of TNF-α (1286 ± 113 pg/ml) and IL-12 (25 ± 0.9 pg/ml), as previously reported [19]. Peptide and adjuvant-free DCm showed no production of TNF-α or IL-12, either in culture or after intraperitoneal inoculation into mice (data not shown) [18, 19].

### B16OVA transplantation into DCm-peptide vaccinated and NV mice

C57BL/6 female mice (*n =* 5) received a single injection of DCm-peptide vaccines (10^6^ cells) in the peritoneal cavity or saline (NV mice) for 7 days. Mice were B16F10 transplanted with a single injection of 5 × 10^5^ B16OVA cells in the peritoneal cavity. On day 14 after B16OVA transplantation and before mice were killed, we collected and processed serum in the first 50 min and stored it at −80°C until cytokine analysis by FACS. On the same day, we collected spleens, transplanted melanoma, livers and lungs of DCm-peptide vaccinated and NV mice to photograph organs and metastases. Spleens and recovered melanoma were also processed for FACS analysis in the following 2 h after their isolation. Melanoma size was measured with a calliper and expressed in mm^2^ by multiplication of diametrically perpendicular measurements. Results are expressed as the mean ± SD.

### Immunohistochemistry

NV mice were sacrificed on days 0, 7 and 14 after B16OVA melanoma transplantation. The most common metastatic organs (liver, spleen, kidney, adrenal glands, liver and lungs) were resected, sectioned, and fixed by immersion in 4% formaldehyde for 24 h. Organs were subsequently embedded in paraffin, processed, and sections stained with hematoxylin–eosin) and immunohistochemical analysis of lymphocyte markers performed as previously described [10]. Primary monoclonal antibodies (Dako, Carpinteria, CA, USA) used were against the following antigens, CD4 (clone 4B12), CD8 (clone c8/144B), CD23 (clone DAK-CD23), CD45 (clone 2B11 + PD7/26), CD56 (clone 123C3) and CD68 (clone KP1) and visualized as described [10].

### Adhesion of B16OVA melanoma to plates

Recovered melanoma after DCm-peptide vaccination or not (NV mice) and transplantation into mice was minced, disaggregated and passed through a 70-μm strainer to obtain a single cell suspension. Cells were counted and seeded into six-well plates at 5 × 10^6^ cells/ml. After 2 h, culture medium was removed and replaced with fresh medium and cells were allowed to grow for 16 h. Cells were detached and viable cells quantified after staining with Trypan blue. B16OVA melanoma not transplanted into mice was also seed into six-well plates at the same concentration and served as a control with 100% adherence. Results were expressed as percentages of viable cells compared to total cells seeded. B16OVA recovered from NV mice had the same percentage of adherent cells as non-transplanted B16OVA melanoma.

### FACS analysis of spleens, melanoma, intracellular IFN-γ staining and cytokine measurements

Cell surface markers of bone-marrow-derived DCs, spleens or recovered melanoma from mice vaccinated with DCm-peptides or NV mice, transplanted with B16OVA, were analysed by FACS using the following antibodies: anti-CD4-PE, anti-CD8α-PE, anti-CD49b-PE, anti-F4/80-PE, anti-CD11b-APC, anti-CD11c-PE, anti-MHC-II-APC, anti-CD40-PE, anti-CD83-APC and anti-CD86-V450 (Miltenyi Biotech). Recovered melanoma cells were also analysed for apoptosis in the following hour after their isolation by FACS using two reported products for apoptosis, Annexin-V conjugated with APC fluorochrome and 7-AAD (7-amino-actinomycin D) (BD-Biosciences, San Jose, CA, USA). Mice sera or supernatants of DCm-peptide vaccines were used to quantify cytokines using the CBA kit (Becton Dickinson, Palo Alto, CA, USA). Samples were analysed in triplicate and results were expressed as the mean ± SD of two separate experiments. For measuring of intracellular IFN-γ, spleen cells were cultured in 96-well plates (5 × 10^6^ cells/ml) and stimulated with B16OVA extract (50 μg/ml) (B16OVA_ext_), LLO_91–99_ or OVA_257–264_ peptides (50 μM) for 5 h in the presence of brefeldin A [38]. Next, cells were surface labelled for CD4 or CD8, fixed and permeabilised with cytofix/cytoperm kit to measure intracellular IFN-γ (BD Biosciences). After sample acquisition, data were gated for CD4^+^ or CD8^+^ events, and the percentages of these cells expressing IFN-γ were determined. Results were corrected according to the percentages of total CD4^+^ or CD8^+^ positive cells. Data were analysed using FlowJo software (Treestar, Ashland, OR, USA).

### Frequencies of LLO or OVA-peptide specific CD8^+^ T cells

To confirm the frequency of LLO_91–99_ or OVA_257–264_-specific CD8 T cells producing IFN-γ, we used recombinant soluble dimeric mouse H-2K^b^:Ig fusion protein (DimerX I; BD Biosciences). LLO_91–99_ or OVA_257–264_ peptides (40 μM) were preincubated with PE-conjugated H-2K^b^:Ig (1 μM) in PBS, at 37°C for 16 h as previously described [17]. Spleen cells (2 × 10^7^/ml) were incubated with IFN-γ and CD8 antibodies and the staining cocktail mix described above for 10 min at 4°C. Percentages of CD8^+^ gated cells were expressed as the mean ± SD of triplicate samples (*P* < 0.05). Data were analysed using FlowJo software.

### Isolation of TILs from transplanted melanoma

B16OVA melanoma was recovered from mice vaccinated with DCm-peptide or not (NV mice) with a single dose of vaccine for 7 days, followed by B16OVA transplantation for 14 days. At the end of melanoma transplantation, we processed melanoma in the first hour after isolation. Melanoma processing included mincing, disaggregating, passing through a 70-μm strainer and isolating TILs by centrifugation over a Ficoll gradient at a 1.077 g/ml density (Histopaque-1077; Sigma–Aldrich, St Louis, MO, USA). We recovered TILs in the interphase gradient, while collecting melanoma cells in pellets. In control samples, we performed an enzymatic digestion with 10% fetal calf serum and collagenase IV (200 U/ml) before Ficoll gradient centrifugation, compared with samples without enzymatic digestion. We observed no differences in the number of TILs recovered in samples digested or not digested with collagenase. Cells were stained with CD4-FITC, CD8-PE, CD25-V459, CD14-APC, CD11b-FITC or CD49b-PE antibodies and analysed by FACS.

### Statistical analysis

For statistical analysis, the Student's *t* test was applied. *P* ≤ 0.05 was considered significant using GraphPad for graphic presentation.

## References

[1] González-Vela MC, Val-Bernal JF, González-López MA, Novell M, Fernandez-Llaca H (2008). Collision of pigmented benign tumours: a possible simulator of melanoma. Acta Derm. Venereol.

[2] Boyano MD, Garcia de Galdeano A, Smith-Zubiaga I, Cavañete ML (1997). IL-2 treatment of B16F10 melanoma cells stimulates metastatic colonization in the liver. Anticancer Res.

[3] Bathia S, Tykodi SS, Thompson JA (2009). Treatment of metastatic melanoma: an overview. Oncology.

[4] Akiyama Y, Tanosaki R, Inoue N, Shimada M, Hotate Y, Yamamoto A, Yamazaki N, Kawashima I, Nukaya I, Takesako K, Maruyama K, Takaue Y, Yamaguchi K (2005). Clinical response in Japanese metastatic melanoma patients treated with peptide cocktail-pulsed dendritic cells. J. Trans. Med.

[5] Oshita C, Takikawa M, Kume A, Miyata H, Ashizawa T, Iizuka A, Kiyohara Y, Yoshikawa S, Tanosaki R, Yamazaki N, Yamamoto A, Takesako K, Yamaguchi K (2012). Dendritic cell-based vaccination in metastatic melanoma patients: Phase II clinical trial. Oncol Rep.

[6] Wimmers F, Aarntzen EH, Schreibelt G, Jacobs JF, Ja Punt C, Figdor CG, De Vries IJ (2014). Early predictive value of multifunctional skin-infiltrating lymphocytes in anticancer immunotherapy. Oncoimmunology.

[7] de Rosa F, Ridolfi L, Ridolfi R, Gentili G, Valmorri L, Nanni O, Petrini M, Fiammenghi L, Granato AM, Ancarani V, Pancisi E, Soldati V, Cassan S (2014). Vaccination with autologous dendritic cells loaded with autologous tumor lysate or homogenate combined with immunomodulating radiotherapy and/or preleukapheresis IFN-α in patients with metastatic melanoma: a randomised “proof-of-principle” phase II study. J. Transl. Med.

[8] Skoberne M, Yewdall A, Bahjat KS, Godefroy E, Lauer P, Lemmens E, Liu W, Luckett W, Leong M, Dubensky TW, Brockstedt DG, Bhardwaj N (2008). KBMA Listeria monocytogenes is an effective vector for DC-mediated induction of antitumor immunity. J. Clin. Invest.

[9] Wood LM, Guirnalda PD, Seavey MM, Paterson Y (2008). Cancer immunotherapy using Listeria monocytogenes and listerial virulence factors. Immunol. Res.

[10] Bronchalo-Vicente L, Rodriguez-Del Rio E, Freire J, Calderon-Gonzalez R, Frande Cabanes E, Gomez-Roman J, Fernandez-Llaca H, Yañez-Diaz S, Alvarez-Dominguez C (2015). A novel therapy for melanoma developed in mice: transformation of melanoma into dendritic cells with Listeria monocytogenes. PLoS One.

[11] Le DT, Wang-Gillam A, Picozzi V, Greten TF, Crocenzi T, Springett G, Morse M, Zeh H, Cohen D, Fine RL, Onners B, Uram JN, Laheru DA (2015). Safety and survival with GVAX pancreas prime and Listeria monocytogenes-expressing mesothelin (CRS-207) boost vaccines for metastatic pancreatic cancer. J. Clin. Oncol.

[12] Kim SH, Castro F, Paterson Y, Gravekamp C (2009). High efficacy of a Listeria-based vaccine against metastatic breast cancer reveals a dual mode of action. Cancer Res.

[13] Maciag PC, Radulovic S, Rothman J (2009). The first clinical used of live-attenuated Listeria monocytogenes vaccine: a Phase I safety study of Lm-LLO-E7 in patients with advanced carcinoma of cervix. Vaccine.

[14] Sun R, Liu Y (2013). Listeriolysin O as a strong immunogenic molecule for the development of new anti-tumor vaccines. Hum. Vaccin. Immunother.

[15] Kono M, Nakamura Y, Suda T, Uchijima M, Tsujimura K, Nagata T, Giermasz AS, Kalinski P, Nakamura H, Chida K (2012). Enhancement of protective immunity against intracellular bacteria using type-1 polarized dendritic cell (DC) vaccine. Vaccine.

[16] Rodriguez-Del Rio E, Marradi M, Calderon-Gonzalez R, Frande-Cabanes E, Penades S, Petrovsky N, Alvarez-Dominguez C (2015). A gold-glyconanoparticle carrying a listeriolysin o peptide and formulated with Advax^™^ delta inulin adjuvant induces robust T-cell protection against Listeria infection. Vaccine.

[17] Calderon-Gonzalez R, Frande-Cabanes E, Bronchalo-Vicente L, Lecea-Cuello MJ, Pareja E, Bosch-Martinez A, Fanarraga ML, Yañez-Diaz S, Carrasco-Marin E, Alvarez-Dominguez C (2014). Cellular vaccines in listeriosis: role of the Listeria antigen GAPDH. Front Cell Infect Microbiol.

[18] Calderon-Gonzalez R, Frande-Cabanes E, Tobes R, Pareja E, Alaez-Alvarez L, Alvarez-Dominguez C (2015). A dendritic cell targetted vaccines loaded with a glyceraldehyde-3-phosphate-dehydrogenase peptide confers wide protection to listeriosis in susceptible and resistant mice. J Vaccines Vaccin.

[19] Calderon-Gonzalez R, Tobes R, Pareja E, Frande-Cabanes E, Alaez-Alvarez L, Petrovsky N, Alvarez-Dominguez C (2015). Identification and characterisation of T-cell epitopes for incorporation into dendritic cell-delivered *Listeria* vaccines. J. Immunol. Methods.

[20] Patyar S, Joshi R, Prasad Byrav DS, Prakash A, Medhi B, Das BK (2010). Bacteria in cancer therapy: a novel experimental strategy. J. Biomed. Sci.

[21] Balch CM, Gershenwald JE, Soong SJ, Thompson JF, Atkins MB, Byrd DR, Buzaid AC, Cochran AJ, Coit DG, Ding S, Eggermont AM, Flaherty KT, Gimotty PA (2009). Final versión of 2009 AJCC melanoma stagins and classification. J. Clin. Oncol.

[22] Ochoa MC, Fioravanti J, Rodriguez I, Hervas-Stubbs S, Azpilikueta A, Mazzolini G, Gúrpide A, Prieto J, Pardo J, Berraondo P, Melero I (2013). Antitumor immunotherapeutic and toxic properties of an HDL-conjugated chimeric IL-15 fusion protein. Cancer Res.

[23] Morales-Kastresana A, Sanmamed MF, Rodriguez I, Palazon A, Martinez-Forero I, Labiano S, Hervas-Stubbs S, Sangro B, Ochoa C, Rouzaut A, Azpilikueta A, Bolaños E, Jure-Kunkel M (2013). Combined immunostimulatory monoclonal antibodies extend survival in an aggressive transgenic hepatocellular carcinoma mouse model. Clin. Cancer Res.

[24] Taniguchi S, Takeoka M, Ehara T, Hashimoto S, Shibuki H, Yoshimura N, Shigematsu H, Takahashi K, Katsuki M (2001). Structural fragility of blood vessels and peritoneum in calponin-h1 deficient mice, resulting in an enhanced haematogeneous metastasis and peritoneal dissemination of malignant tumour cells. Cancer Res.

[25] Hashimoto S, Takeoka M, Taniguchi S (2003). Suppression of peritoneal dissemination through protecting mesothelial cells from retraction by cancer cells. Int. J. Cancer.

[26] Wallecha A, Wood L, Pan ZK, Maciag PC, Shahabi V, Paterson Y (2013). Listeria monocytogenes-derived listeriolysin O has pathogen-associated molecular pattern-like properties independent of its hemolytic ability. Clin. Vaccine Immunol.

[27] Wood LM, Paterson Y (2014). Attenuated Listeria monocytogenes: a powerful and versatile vector for the future of tumour immunotherapy. Front. Cell. Infect. Microbiol.

[28] Jangi SM, Ruiz-Larrea MB, Nicolau-Galmés F, Andollo N, Arroyo-Berdugo Y, Ortega-Martinez I, Diaz-Perez JL, Boyano-Lopez MD (2008). Terfenadine-induced apoptosis in human melanoma cells is mediated through Ca2+ homeostasis modulation and tyrosine kinase activity, independently of H1 histamine receptors. Carcinogenesis.

[29] Nicolau-Galmés F, Asumendi A, Alonso-Tejerina E, Pérez-Yarza G, Jangi SM, Gardeazabal J, Arroyo-Berdugo Y, Careaga JM, Díaz-Ramón JL, Apraiz A, Boyano MD (2011). Terfenadine induces apoptosis and autophagy in melanoma cells through ROS-dependent and -independent mechanisms. Apoptosis.

[30] Nitcheu-Tefit J, Dai MS, Critchley-Thorne R, Ramirez-Jimenez F, Xu M, Conchon S, Ferry N, Stauss HJ, Vassaux G (2007). Listeriolysin O expressed in a bacterial vaccine suppresses CD4+CD25high regulatory T cell function *in vivo*. J. Immunol.

[31] Viguier M, Lemaitre F, Verola O, Gorochov G, Dubertret L, Bachelez H, Kourilsky P, Ferradini L (2004). Foxp3 expressing CD4 + CD25high regulatory T cells are overrepresented in human metastatic melanoma lymph nodes and inhibit the function of infiltrating T cells. J. Immunol.

[32] Guekara NO, Jacobs T, Chakraborty T, Weiss S (2006). The cholesterol-dependent cytolysin lysteriolysin O aggregates rafts via oligomerization. Cell. Microbiol.

[33] Javia LR, Rosenberg SA (2003). CD4+CD25high suppressor lymphocytes in the circulation of patients immunized against melanoma antigens. J. Immunother.

[34] Mitchell DA, Batich KA, Gunn MD, Huang MN, Sanchez-Perez L, Nair SK, Congdon KL, Reap EA, Archer GE, Desjardins A, Friedman AH, Friedman HS, Herndon JE (2015). Tetanous toxoid and CCL3 improve dendritic cell vaccines in mice and gliobastoma patients. Nature.

[35] Montes M, Rufer N, Appay V, Reynard S, Pittet MJ, Speiser DE, Guillaume P, Cerottini JC, Romero P, Leyvraz S (2005). Optimum *in vitro* expansion of human antigen-specific CD8+ T cells for adoptive transfer therapy. Clin. Exp. Immunol.

[36] Ellebaek E, Iversen TZ, Junker N, Donia M, Engell-Noerregaard L, Met Ö, Hölmich LR, Andersen RS, Hadrup SR, Andersen MH, thor Straten P, Svane IM (2012). Adoptive cell therapy with autologous tumor infiltrating lymphocytes and low-dose interleukin-2 in metastatic melanoma patients. J. Trans. Med.

[37] Dobrzanski MJ, Reome JB, Dutton RW (1999). Therapeutic effects of tumor-reactive type 1 and type 2 CD8+ T cell subpopulations in established pulmonary metastases. J. Immunol.

[38] Carrasco-Marín E, Rodriguez-Del Rio E, Frande-Cabanes E, Tobes R, Pareja E, Lecea Cuello MJ, Ruiz-Saez M, Madrazo-Toca F, Hölscher C, Alvarez-Dominguez C (2012). Phagosomes induced by cytokines function as anti-Listeria vaccines: novel role for functional compartmentalization of STAT-1 protein and cathepsin-D. J. Biol. Chem.

